# Comparative Outcomes of Milligan-Morgan (Open) Versus Ferguson (Closed) Hemorrhoidectomy: A Retrospective Study

**DOI:** 10.7759/cureus.75012

**Published:** 2024-12-03

**Authors:** Syed Saad Ali Chishti, Abdal Niaz, Muhammad Kashif, Wajid Ali

**Affiliations:** 1 Surgery, Pakistan Institute of Medical Sciences, Islamabad, PAK; 2 Surgery, Cavan General Hospital, Cavan, IRL; 3 General Surgery, Rehman Medical Institute, Peshawar, PAK; 4 Gastroentrology, Lady Reading Hospital, Peshawar, PAK; 5 Surgery, Hayatabad Medical Complex, Peshawar, PAK

**Keywords:** ferguson hemorrhoidectomy, hemorrhoidectomy, milligan-morgan hemorrhoidectomy, open hemorrhoidectomy, outcomes

## Abstract

Introduction

The debate remains unresolved about whether an open (Milligan-Morgan) or closed (Ferguson) approach is superior for hemorrhoidectomy. Advocates from both groups state that each has its own set of advantages and disadvantages. In light of this, we intend to share our experience by comparing the two in terms of their post-operative outcomes. This study aims to compare open (Milligan-Morgan) and closed (Ferguson) hemorrhoidectomy in terms of post-operative outcomes.

Materials and methods

This retrospective study was carried out at the Department of Surgery, Pakistan Institute of Medical Sciences, Islamabad, Pakistan. A total of 137 patients who underwent hemorrhoidectomy from January 1, 2022, to May 31, 2024, were enrolled. Post-operative outcomes were noted in terms of surgical site infection (SSI), excessive bleeding, and visual analog scale (VAS) pain score. Data were analyzed using IBM SPSS Statistics for Windows, Version 25 (Released 2017; IBM Corp., Armonk, NY, USA).

Results

A total of 137 patients were enrolled in the study, with 93 (67.9%) undergoing Milligan-Morgan (open) hemorrhoidectomy and 44 (32.1%) undergoing the Ferguson (closed) procedure. The mean age in the open group was 46.87 ± 10.79 years, compared to 44.59 ± 9.06 years in the closed group. Male participants comprised 59 (63.4%) in the open group and 19 (43.2%) in the closed group. SSI was observed in 32 patients (23.3%); 22 (68.8%) were from the open group, and 10 (31.3%) were from the closed group (p-value, 0.905). Hemorrhage was recorded in 34 patients (15.3%), with 21 (61.8%) in the open group and 13 (38.2%) in the closed group (p-value, 0.378). The mean post-operative VAS pain score was 3.76 ± 1.79 for the open technique versus 4.07 ± 1.37 for the closed technique, respectively (p-value, 0.321).

Conclusion

Though the SSI and hemorrhage rates were higher with the Milligan-Morgan technique than with the Ferguson technique, the mean post-operative VAS score was also higher with the Milligan-Morgan procedure. However, none of the differences was found to be statistically significant.

## Introduction

The architecture of the anal canal is maintained by the cushioning effect of the submucosa, which surrounds vascular tissue from all directions. Loss of this cushioning phenomenon leads to prolapse of vascular tissue, resulting in a clinical condition called hemorrhoids [1]. Depending on the location with respect to the dentate line, hemorrhoids can be external (below the dentate line) or internal (above the dentate line). Both types differ in the nature and severity of the symptoms [2].

Prolonged constipation, excessive straining, chronic cough, and conditions leading to increased intraperitoneal pressure are some of the significant risk factors for the development of hemorrhoids [3]. The type and severity of symptoms vary with the degree of engorgement and prolapse, with mild or no symptoms in grade 1 and 2 hemorrhoids, while bleeding, itching, and a burning sensation occur in the later stages of the disease process (grade 3 and 4 hemorrhoids) [4]. Common complications of hemorrhoids include anemia and thrombosis [5]. Treatment of hemorrhoids is directed towards the severity of symptoms and grades of hemorrhoids. Grade 1 and 2 hemorrhoids are often managed conservatively, while surgery is advocated for grade 3 and 4 hemorrhoids or hemorrhoids complicated by thrombosis or anemia [6].

Several surgical techniques have been developed over the years for hemorrhoidectomy. Historically, open hemorrhoidectomy (Milligan-Morgan technique) has been the most commonly performed procedure for the treatment of hemorrhoids [7]. It is still the most frequently advocated procedure, particularly in resource-limited settings. Like other traditional procedures, post-operative pain and delayed recovery are some of the major limitations of the procedure [8]. With growing trends toward the implementation of interventions with limited post-operative morbidities and early resumption of daily activities, the closed technique (Ferguson technique) has been in practice for quite some time for the treatment of hemorrhoids, believed to address the issues associated with the Milligan-Morgan technique [9]. However, neither technique has been previously evaluated in local settings. Hence, the study was carried out to compare Milligan-Morgan (open) and Ferguson (closed) techniques for post-operative outcomes in the treatment of hemorrhoids.

## Materials and methods

This descriptive comparative study design was chosen to analyze patient records at the Department of Surgery, Pakistan Institute of Medical Sciences, Islamabad, Pakistan.

Male and female participants in the age range of 20-80 years who underwent surgical treatment for hemorrhoids during the period from January 1, 2022, to May 31, 2024, were enrolled after approval from the Ethical Review Committee (Approval No.: 279). Patients with incomplete records, recurrent hemorrhoids, anal fistula, external hemorrhoids, and comorbid or associated anorectal disorders compromising surgery or outcomes were excluded. Patients were recruited using a non-probability consecutive sampling technique. The sample size was 137, which was calculated using the WHO (World Health Organization) sample calculator, taking a 9.85% overall rate of surgical infection after hemorrhoidectomy, with a 5% margin of error and a 95% confidence interval [10].

Hemorrhoids were confirmed clinically using a proctoscope. Patients with grade 3 and 4 hemorrhoids were considered significant, defined as protruding hemorrhoids that are manually reducible (grade 3) and permanently prolapsed hemorrhoids (grade 4). Surgical site infection (SSI) was confirmed clinically by the presence of serosanguinous discharge from the surgical wound site within 14 post-operative days. Excessive bleeding was defined as soaking more than five dressings within 24 hours after surgery. Post-operative pain was measured using a visual analog scale (VAS) score 24 hours after surgery. A VAS score greater than 4 was considered significant.

The records of patients satisfying the selection criteria were retrieved from the hospital management information system. Baseline sociodemographic and clinical parameters of interest, such as age, gender, and body mass index (BMI) calculated using the formula weight in kilograms/height in meters squared, any comorbidities like diabetes mellitus, hypertension, and coronary artery disease, operative details, and complications, were retrieved.

Patients were assigned to group A (Milligan-Morgan - open) and group B (Ferguson - closed) based on the type of surgery. All surgeries were performed under general anesthesia. The choice of surgery was decided based on patient preference and clinical factors, such as the grade of hemorrhoids. Standard pre-operative measures were taken, including bowel preparation and antibiotic care. All procedures were carried out in the lithotomy position. In group A, dissection of the protruding veins was carried out after the application of three clamps, followed by ligation of the pedicles with absorbable sutures. Hemostasis was secured, and a dressing was applied to the wound. In group B, access to hemorrhoidal veins was achieved through the application of a Ferguson retractor, followed by compression of the veins at the base. The incision was made around the column, and the pedicle was dissected off. The remnant was ligated with an absorbable suture. To reduce the risk of recurrence, another deep suture was applied at the top of the anorectal ring. Anal packing was placed and removed every six hours until there was no soaking of the packs with blood. Standard post-operative care was given to all patients as per hospital protocol, comprising antibiotics and pain management. The number of pack changes during the first 24 hours was noted. Patients were asked to report the presence and severity of post-operative pain 24 hours after surgery. All patients were followed for two weeks. The presence of SSI was noted as per operational definitions.

Data analysis was carried out using IBM SPSS Statistics for Windows, Version 25 (Released 2017; IBM Corp., Armonk, NY, USA). Continuous data were presented as means ± SD or median (interquartile range, IQR) after checking the normality of the data with the Shapiro-Wilk test. Normally distributed data were compared using the Student t-test, and non-normally distributed data were compared using the Mann-Whitney U test. A p-value of ≤0.05 was considered significant.

## Results

A total of 137 patients were enrolled, 93 (67.9%) of whom underwent Milligan-Morgan (group A), and 44 (32.1%) underwent the Ferguson technique (group B). As illustrated in Table 1, the mean age of the participants in groups A and B was 46.87 ± 10.79 and 44.59 ± 9.061 years, respectively (p-value, 0.227). The mean BMI in group A was 23.56 ± 2.89, versus 25.48 ± 2.10 kg/m^2^ (p-value < 0.001).

**Table 1:** Mean and standard deviations of patients according to various parameters, n = 137 (Milligan-Morgan (group A) = 93; Ferguson (group B) = 44) p-value of ≤0.05 was considered significant. BMI, Body mass index; VAS, Visual analog scale

Parameters	Group	Mean	Standard deviation	p-value
Age (years)	Milligan-Morgan	46.87	10.79	0.227
	Ferguson	44.59	9.061	
BMI (kg/m^2^)	Milligan-Morgan	23.56	2.89	0.000
	Ferguson	25.48	2.10	
Post-operative pain (VAS)	Milligan-Morgan	3.76	1.79	0.321
	Ferguson	4.07	1.37	
Disease duration (months)	Milligan-Morgan	13.02	3.84	0.002
	Ferguson	15.20	3.78	

As presented in Table 2, patients older than 50 years were 29 (74.4%) in group A and 10 (25.6%) in group B (p-value, 0.306). Male patients were 59 (75.6%) in group A and 19 (24.4%) in group B (p-value, 0.025). A BMI >24.0 kg/m^2^ in group A was recorded in 26 (54.2%) and 22 (45.8%) in group B (p-value, 0.012). The p-value for the difference in disease duration between the two groups was 0.001.

**Table 2:** Frequencies and percentages of patients according to various characteristics, n = 137 (Milligan-Morgan (group A) = 93; Ferguson (group B) = 44) p-value of ≤0.05 was considered significant. Significant p-values are indicated by an asterisk (*). BMI, Body mass index; CAD, Coronary artery disease; DM, Diabetes mellitus

Parameters	Subgroups	Group		Chi-square p-value
		Milligan-Morgan (group A)	Ferguson (group B)	
Age (years)	50 or below	64	34	0.306
		65.3%	34.7%	
	More than 50	29	10	
		74.4%	25.6%	
Gender	Male	59	19	0.025
		75.6%	24.4%	
	Female	34	25	
		57.6%	42.4%	
BMI (kg/m^2^)	24.0 or below	67	22	0.012*
		75.3%	24.7%	
	More than 24.0	26	22	
		54.2%	45.8%	
Disease duration (months)	15 or below	67	19	0.001*
		77.9%	22.1%	
	More than 15	26	25	
		51.0%	49.0%	
Disease grade	Grade 3	42	21	0.778
		66.7%	33.3%	
	Grade 4	51	23	
		68.9%	31.1%	
Comorbidities	DM	18	8	0.749
		69.2%	30.8%	
	Hypertension	25	15	
		62.5%	37.5%	
	CAD	16	5	
		76.2%	23.8%	
	None	34	16	
		68.0%	32.0%	

Table 3 compares outcomes in both groups. The rate of SSI in the Milligan-Morgan procedure was 22 (23.7%), compared to 10 (22.7%) in the Ferguson technique. Hemorrhage was recorded in 21 (22.6%) in the Milligan-Morgan group and 13 (29.5%) in the Ferguson technique. Pain (VAS > 4) was observed in 61 patients (65.6%) with Milligan-Morgan and 28 (63.6%) with the Ferguson technique. The p-value for the SSI rate was 0.905, which is >0.05, hence statistically not significant. Similarly, the p-values for comparison between hemorrhage and pain (VAS > 4) were 0.378 and 0.823, respectively.

**Table 3:** Frequencies and percentages of outcomes in Milligan-Morgan versus Ferguson hemorrhoidectomy, n = 137 (Milligan-Morgan (group A) = 93; Ferguson (group B) = 44) p-value of ≤0.05 was considered significant. VAS, Visual analog scale

Outcomes	Group	Subgroup	Frequency	Percent	p-value
Surgical site infection	Milligan-Morgan	Yes	22	23.7	0.905
		No	71	76.3	
	Ferguson	Yes	10	22.7	
		No	34	77.3	
Hemorrhage	Milligan-Morgan	Yes	21	22.6	0.378
		No	72	77.4	
	Ferguson	Yes	13	29.5	
		No	31	70.5	
Pain (VAS > 4)	Milligan-Morgan	Yes	61	65.6	0.823
		No	32	34.4	
	Ferguson	Yes	28	63.6	
		No	16	36.4	

Logistic regression analysis was applied to assess the association between outcomes and determining factors, as shown in Tables 4-6. No statistically significant association was recorded.

**Table 4:** Binary logistic regression analysis for predicting surgical site infection, n = 137 (Milligan-Morgan (group A) = 93; Ferguson (group B) = 44) p-value of ≤0.05 was considered significant. BMI, Body mass index; CAD, Coronary artery disease; SE, Standard error; B, Beta coefficient; Exp(B), Exponentiated beta coefficient; Sig, Significance

Group	Variables	B	SE	Wald	Sig	Exp(B)
Milligan-Morgan	Age (≤50 years)	0.665	0.552	1.449	0.229	1.944
	Gender, male	-0.077	0.549	0.020	0.889	0.926
	BMI (≤24.0 kg/m^2^)	-0.039	0.602	0.004	0.948	0.962
	Duration ≤15 months	0.909	0.563	2.606	0.106	2.482
	Grade 3 disease	-0.878	0.539	2.655	0.103	0.416
	Diabetes	-0.554	0.671	0.683	0.408	0.574
	Hypertension	0.273	0.709	0.148	0.700	1.314
	CAD	-0.171	0.768	0.050	0.824	0.843
Ferguson	Age (≤50 years)	-20.40	1161.2	0.000	0.999	0.000
	Gender, male	0.787	0.985	0.640	0.424	2.198
	BMI (≤24.0 kg/m^2^)	-1.448	1.009	2.060	0.151	0.235
	Duration ≤15 months	-0.085	0.980	0.008	0.931	0.918
	Grade 3 disease	-0.274	0.953	0.083	0.774	0.760
	Diabetes	0.062	1.360	0.002	0.963	1.064
	Hypertension	-1.052	1.016	1.073	0.300	0.349
	CAD	-1.842	1.491	1.526	0.217	0.158

**Table 5:** Binary logistic regression analysis for predicting hemorrhage, n = 137 (Milligan-Morgan (group A) = 93; Ferguson (group B) = 44) p-value of ≤0.05 was considered significant. BMI, Body mass index; CAD, Coronary artery disease; SE, Standard error; B, Beta coefficient; Exp(B), Exponentiated beta coefficient; Sig, Significance

Group	Variables	B	SE	Wald	Sig	Exp(B)
Milligan-Morgan	Age (≤50 years)	-0.860	0.656	1.719	0.190	0.423
	Gender, male	-1.276	0.699	3.337	0.068	0.279
	BMI (≤24.0 kg/m^2^)	1.140	0.584	3.812	0.051	3.128
	Duration ≤15 months	-0.738	0.731	1.017	0.313	0.478
	Grade 3 disease	-0.002	0.555	0.000	0.998	0.998
	Diabetes	-0.311	0.774	0.162	0.688	0.733
	Hypertension	-0.096	0.713	0.018	0.892	0.908
	CAD	-0.914	0.799	1.309	0.253	0.401
Ferguson	Age (≤50 years)	1.906	1.017	3.514	0.061	6.723
	Gender, male	1.883	1.029	3.348	0.067	6.572
	BMI (≤24.0 kg/m^2^)	0.051	0.838	0.004	0.951	1.053
	Duration ≤15 months	-1.500	0.907	2.734	0.098	0.223
	Grade 3 disease	-0.430	0.779	0.305	0.581	0.650
	Diabetes	-1.076	1.045	1.061	0.303	0.341
	Hypertension	0.741	1.023	0.524	0.469	2.097
	CAD	-0.109	1.196	0.008	0.928	0.897

**Table 6:** Binary logistic regression analysis for predicting pain, n = 137 (Milligan-Morgan (group A) = 93; Ferguson (group B) = 44) p-value of ≤0.05 was considered significant. Significant p-values are indicated by an asterisk (*). BMI, Body mass index; CAD, Coronary artery disease; SE, Standard error; B, Beta coefficient; Exp(B), Exponentiated beta coefficient; Sig, Significance

Group	Variables	B	SE	Wald	Sig	Exp(B)
Milligan-Morgan	Age (≤50 years)	-0.371	0.499	0.552	0.457	0.690
	Gender, male	-0.164	0.499	0.109	0.742	0.848
	BMI (≤24.0 kg/m^2^)	-1.256	0.515	5.941	0.015*	0.285
	Duration ≤15 months	-0.264	0.523	0.255	0.614	0.768
	Grade 3 disease	-0.290	0.480	0.364	0.546	0.749
	Diabetes	-0.302	0.635	0.226	0.634	0.739
	Hypertension	-0.355	0.585	0.368	0.544	0.701
	CAD	-0.465	0.712	0.428	0.513	0.628
Ferguson	Age (≤50 years)	1.654	1.182	1.957	0.162	5.226
	Gender, male	-1.005	0.795	1.598	0.206	0.366
	BMI (≤24.0 kg/m^2^)	0.228	0.766	0.089	0.766	1.256
	Duration ≤15 months	0.392	0.791	0.246	0.620	1.480
	Grade 3 disease	-0.210	0.783	0.072	0.789	0.811
	Diabetes	-1.788	1.244	2.064	0.151	0.167
	Hypertension	-0.336	0.807	0.174	0.677	0.714
	CAD	-1.598	1.372	1.357	0.244	0.202

## Discussion

Out of the total 137 patients, SSI was observed in 32 patients (23.3%); 22 (68.8%) belonged to the Milligan-Morgan group, and 10 (31.3%) belonged to the Ferguson group. The p-value for the difference in SSI between both groups was statistically insignificant. In a study by Farid et al., the frequency of SSI was recorded in 6.7% of patients with the Ferguson technique, compared to 10.9% with the Milligan-Morgan technique. The overall infection rate in their study was lower than our observation. However, similar to our findings, the infection rate was lower with the closed technique compared to the open [10]. In a single-group study of post-operative outcomes in open hemorrhoidectomy by Barman et al., the percentage of surgical wound infections was 4.0%, which was much lower than our findings [11]. The difference in the observations may be attributed to differences in peri-operative prophylactic measures and risk factors, such as poor nutritional status, for the development of SSI in anorectal surgeries [12]. A study by Rehman et al. reported poor wound healing in open hemorrhoidectomy compared to closed hemorrhoidectomy, with a p-value of 0.001. No definitive conclusion was drawn about the factors leading to poor wound healing with the open technique [13].

Hemorrhage was recorded in 34 patients (15.3%) out of the total 137 operated patients; the proportion of hemorrhage in the Milligan-Morgan (open) technique was 21 (61.8%), compared to 13 (38.2%) in the Ferguson group (closed). Though the rate of hemorrhage was higher with the open technique, the difference was statistically not significant (p-value, 0.378). In a study on 2,840 cases of hemorrhoidectomy, excessive bleeding was observed in 1.9% of cases with the Milligan-Morgan technique and 0.6% of cases with Ferguson hemorrhoidectomy, showing a higher proportion with the open technique [14]. Excessive bleeding requiring blood transfusion was reported in 1.6% of patients who underwent open hemorrhoidectomy. However, no control group was used for comparison in the study [15]. A comparative study on post-hemorrhoidectomy bleeding in Ferguson versus Ligasure showed a statistically significant lower incidence of post-operative bleeding with the Ferguson technique (5.1% versus 11.9%; p-value, 0.015) [16]. Compared to these studies, the hemorrhage rate in our study was higher, which may be due to the small sample size in our study and also due to the different criteria used to define hemorrhage.

The mean post-operative VAS pain score 24 hours after surgery in the open versus closed group was 3.76 ± 1.79 versus 4.07 ± 1.37, with a p-value of 0.321. Patients with a VAS score >4 were 61 (68.5%) in the open group, compared to 28 (31.5%) in the closed group (p-value, 0.823). Though the pain score was higher with the closed technique, the difference was statistically not significant. Post-hemorrhoidectomy pain is challenging to manage. Several pharmacological and surgical modification strategies have been devised to address the suboptimal results leading to prolonged recovery [17]. Pain with traditional excisional techniques is attributed to the interruption of sensitive anal mucosa and internal sphincter, which are disrupted [18]. The closed technique was superior to the open technique in terms of lower post-operative pain, as shown in a study by Singh et al. [19]. Internal sphincterotomy can further reduce pain in the closed technique [20]. A randomized controlled trial by Ali et al. comparing Milligan-Morgan and stapled hemorrhoidectomy for post-operative pain showed that clinically significant pain was reported by 12 patients (13.3%) with stapled hemorrhoidectomy, compared to 69 patients (76.7%) with the Milligan-Morgan technique. The difference was statistically significant (p-value < 0.001) [21].

Overall, the study provided vital insights by comparing traditional hemorrhoidectomy techniques for post-operative complications. However, the study still had certain limitations, such as its retrospective study design. A prospective study would have generated more reliable results. The study was limited to a single center, which hindered the generalizability of the findings. A multicenter study would have overcome this limitation. The results would have been further improved if the sample size had been larger than the current one.

## Conclusions

Both the Milligan-Morgan and Ferguson techniques stand as viable options for hemorrhoidectomy. Though the SSI rate was slightly higher with the Milligan-Morgan technique, the difference was statistically insignificant. A similar conclusion was drawn for hemorrhage and post-operative pain between these two techniques. We can conclude that the open (Milligan-Morgan) and closed (Ferguson) techniques appear to have no significant advantages over each other regarding patient comfort and post-operative complications.
